# The Cold Shock Protein CspB from *Mycobacterium tuberculosis* Binds to MTS0997 sRNA and MTS1338 sRNA as a Dimer

**DOI:** 10.3390/ijms27020663

**Published:** 2026-01-09

**Authors:** Natalia Lekontseva, Alisa Mikhaylina, Polina Pankratova, Alexey Nikulin

**Affiliations:** Institute of Protein Research, Russian Academy of Sciences, Institutskaya 4, 142290 Pushchino, Russianikulin@vega.protres.ru (A.N.)

**Keywords:** *Mycobacterium tuberculosis*, sRNA, RNA chaperone, RNA–protein interactions, cold shock proteins, CspB, α-helix hairpin

## Abstract

RNA chaperones play a crucial role in the biogenesis and function of various RNAs in bacteria. They facilitate the interaction of small regulatory trans-encoded sRNAs with mRNAs, thereby significantly altering the pattern of gene expression in cells. This allows bacteria to respond quickly to changing environmental conditions, such as stress or adaptation to host organisms. Despite the identification of a large number of sRNAs in mycobacteria, none of the most common RNA chaperones have been found in their genomes. We determined the crystal structure of the cold shock protein CspB from *Mycobacterium tuberculosis*. It forms a dimer due to its elongated C-terminal region, which is a hairpin composed of two α-helices. It was also demonstrated that CspB from *M. tuberculosis* exhibits high affinity for MTS0997 sRNA and MTS1338 sRNA from the same organism, which is consistent with classical RNA chaperons such as Hfq and ProQ. Based on the putative RNA chaperone activity of bacterial proteins with cold-shock domains, we propose that CspB from *M. tuberculosis* may be involved in the regulation of mycobacterial pathogenesis through interaction with sRNAs.

## 1. Introduction

Numerous physiological processes in bacteria are regulated by small regulatory RNAs (sRNAs) to remodel their gene expression in response to changing environmental conditions [1]. sRNAs help bacteria to adaptively react to the changing environmental conditions and regulate key stages of pathogenesis [2]. Mycobacteria are known to express small RNAs that regulate protein expression by controlling mRNA stability, processing, and access to ribosome binding sites. These molecules have been shown to be closely linked to bacterial survival under stressful conditions, including the host immune response [3,4]. One of these RNAs, MTS1338 sRNA (DrrS sRNA or ncRv11733 sRNA), is highly expressed during the stationary phase of growth [5] and the dormancy state [6]. *In vitro* experiments demonstrated that its transcription is controlled by the transcriptional regulator DosR and is activated under hypoxic and NO-induced stresses [7], suggesting that MTS1338 may play a role during the stable phase of infection, when host responses confront mycobacterial multiplication more or less successfully. MTS0997 sRNA (Mcr11 or ncRv1264Ac) upregulates several genes required for *Mycobacterium tuberculosis* fatty acid production [8]. MTS0997 is abundantly expressed by *M. tuberculosis* in the lungs of chronically infected mice [9] as well as in hypoxic and non-replicating *M. tuberculosis* [4].

In the majority of bacteria species, trans-encoded sRNAs require RNA chaperones, either Hfq or ProQ, to ensure appropriate sRNA/mRNA base pairing [10,11]. However, experiments to identify the orthologues of the most common RNA chaperones Hfq, ProQ, or CsrA in *M. tuberculosis* have failed, which suggests that mycobacteria must exploit alternative proteins or mechanisms for efficient sRNA/mRNA interactions [12]. Possible alternatives are either mycobacterial sRNAs can interact with their cognate mRNAs independently without the assistance of RNA chaperons, or they have an RNA chaperone that has not been discovered yet [13]. One potential candidate for the role of RNA chaperone may belong to the cold shock protein family Csp. Cold shock proteins are multifunctional DNA/RNA binding proteins that are found in 72% of sequenced bacterial genomes [14]. Functionally, bacterial proteins with the Csp domain are mainly transcription and translation factors, and it is also believed that they are involved in the remodeling of RNA structure, that is, they have RNA chaperone activity [15,16,17]. Homologues of Csp are widespread in prokaryotes, acting in response to many different environmental stresses [15,18]. It has been shown that during cold shock, some Csp proteins exhibit their chaperone activity and melt secondary RNA structures, restoring processes such as transcription and translation [19]. The number of Csp proteins varies among bacteria; for example, in *E. coli* nine Csp proteins have been identified, named CspA to CspI, and only some of the homologues have RNA chaperone activity [20]. Two cold shock proteins have been identified in *M. tuberculosis*—CspA and CspB. Both of them contain a β-barrel cold shock domain (Csd) consisting of a three-stranded (β1-β2-β3) N-terminal and a two-stranded (β4-β5) C-terminal sheet. Recently we characterized CspA from *M. tuberculosis* (MtbCspA) and showed that it has a lower affinity for single-stranded RNA compared to single-stranded DNA despite the active involvement of the β3-β4 and β4-β5 loops in interactions with the oligonucleotides [21].

CspB from *M. tuberculosis* (MtbCspB) has a long C-terminal region conserved among mycobacterial proteins and absent from other bacterial Csp proteins. In this study, we report the previously unexplored structure of the MtbCspB protein and its RNA-binding properties, targeting two small RNAs, MTS0997 and MTS1338, which are present only in the genomes of highly pathogenic mycobacteria.

## 2. Results

### 2.1. MtbCspB Forms Dimers

**Figure 1:**
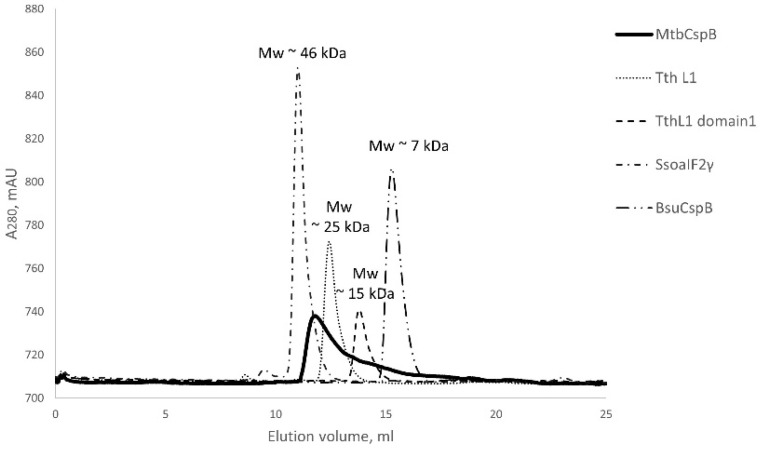
Elution profiles of CspB form *M. tuberculosis* (MtbCspB), gamma subunit of archaeal initiation factor aIF2γ from *Sulfolobus solfataricus* (Sso aIF2γ; PDB ID 4M2L), ribosomal protein L1 from *Thermus thermophilus* (Tth L1; PDB ID 4F9T), isolated domain I of L1 from *T. thermophilus* (Tth L1 domain 1; PDB ID 2OUM) and CspB from *Bacillus subtilis* (BsuCspB; PDB ID 1CSP) (Superdex 75 Increase column 10/300 GL was used). Their molecular weights are indicated on the corresponding peaks.

Based on the AlphaFold prediction, we suggested that MtbCspB could form dimers connecting the C-terminal α-helix hairpins. Under denaturing conditions, MtbCspB had a molecular weight consistent with a monomer—15.9 kDa (Figure S1). However, gel filtration analysis of the protein revealed that MtbCspB preferentially forms dimers, as its peak is located between the peaks of proteins with molecular masses of 25 and 46 kDa (Figure 1). To estimate the molecular mass of MtbCspB we used monomer globular proteins with known crystal structures. The broadening of the protein peak on the chromatogram may be associated with the significant mobility of the N- and C-terminal domains of the protein relative to each other.

### 2.2. The MtbCspB Crystal Structure

**Figure 2:**
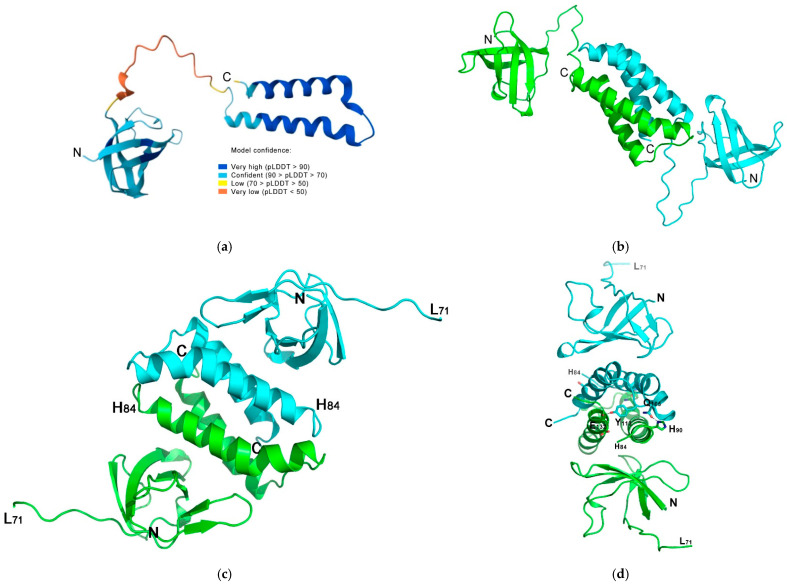
Spatial structure of CspB from *M. tuberculosis*. (**a**) An AlphaFold model (AF-I6WZM9-F1) with coloring according to the model confidence. (**b**) An AlphaFold3 model of the MtbCspB dimer. The two monomers are shown in green and cyan. (**c**) Overall crystal structure of MtbCspB (PDB 9XW3). Two symmetrically related molecules are shown. Residues L71 and H84 mark a break in the protein backbone. (**d**) Side view of the MtbCspB crystal structure. Pairs of amino acids that form intermolecular hydrogen bonds (His90-Gln105 and Tyr113-Glu132) are marked.

The structure of MtbCspB was determined by molecular replacement, and the protein monomer predicted by AlphaFold (AF-I6WZM9-F1, deposited at UNIPROT I6WZM9) was used (Figure 2a). The initial model consisted of an N-terminal Csp domain, a C-terminal double α-helix hairpin and a long unstructured connection of these two parts of the protein. We found one protein monomer in the asymmetric unit of the P3121 cell, divided into the N-terminal Csp domain (residues 2–71) and C-terminal double α-helix hairpin (residues 84–135). The α1-helix (residues 88–110) bended in the region of residues 102–104. No electron density was found for residues 72–83. Analysis of the molecular crystal packing revealed that MtbCspB formed a dimer in the crystal due to tight contacts of the C-terminal parts and organized into a stable bundle of four α-helixes. According to PISA analysis, the buried surface within the bundle was 2440 sq. Å and ΔGint was −20.6 kcal/mol. This α-helical bundle was stabilized by a large number of hydrophobic contacts between the α-helixes. Moreover, four hydrogen bonds formed by the amino acid residues of the α-helixes shielded the ends of the bundle. Thus, the α-helical bundle was ordered in a stable spatial structure that facilitated the organization of the MtbCspB dimer. AlfaFold3 predicted the formation of the protein dimer in the same way, but in an elongated form that can be realized in solution (Figure 2).

### 2.3. MtbCspB Is Able to Bind sRNAs MTS0997 and MTS1338

We determined the affinity of MtbCspB for two of the best-studied sRNAs from *Mycobacterium tuberculosis*—MTS0997 and MTS1338—using surface plasmon resonance (SPR). Both sRNAs were transcribed and purified *in vitro*. The protein was immobilized on the COOH chip and different concentrations of the sRNAs were used as analytes. MtbCspB interacts with the sRNAs with high affinity and does not interact with oligoC RNA, which was used as a control (Table 1, Figure S2) [22,23]. Cold shock proteins are known to bind to uridine-rich RNA so we also tested the protein’s ability to interact with oligoU RNA [22,23]. The affinity of the protein to the oligoU RNA is two orders of magnitude lower.

### 2.4. MtbCspB Modulates the Structure of MTS0997 and MTS1338 sRNAs Differently

**Figure 3:**
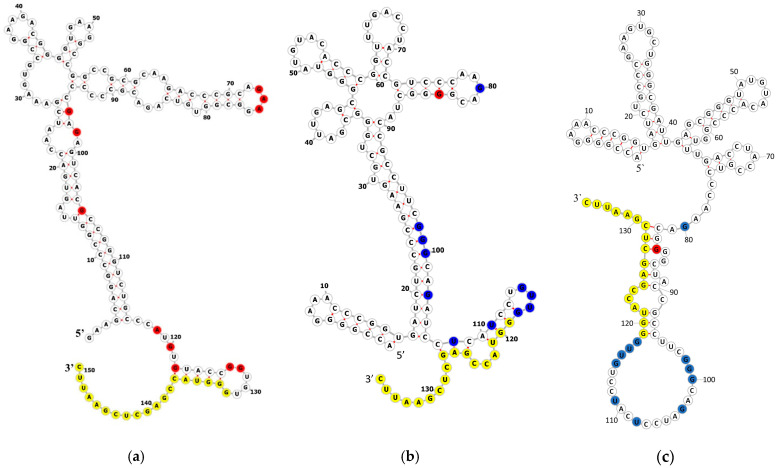

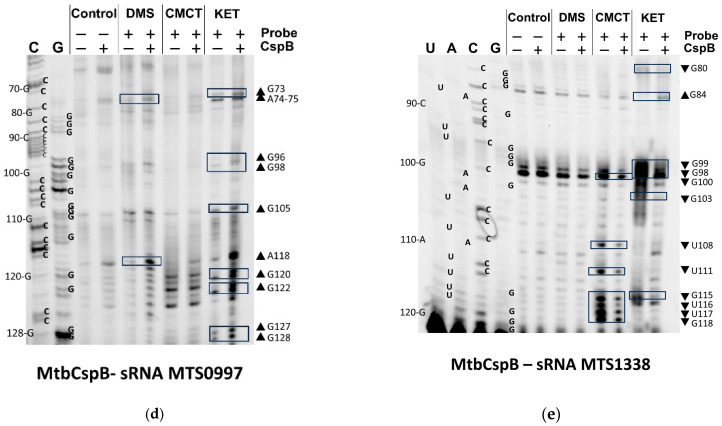
Chemical probing of the RNAs alone and in the presence of MtbCspB. Secondary structures of MTS0997 (**a**) and MTS1338 (**b**,**c**) mRNAs predicted using RNAfold. In case (**c**), the predicted structure is based on chemical probing results. Nucleotides whose reactivity increased with the addition of MtbCspB are shown in red; nucleotides whose reactivity decreased with the addition of MtbCspB are shown in blue; nucleotides complementary to the primer are shown in yellow. (**d**,**e**) Representative gels showing structure probing of MTS0997 and MTS1338 sRNAs using DMS, CMCT, and kethoxal (KET) in the presence and absence of MtbCspB. Unmodified RNAs alone or in the complex with MtbCspB were used as controls. U, A, C, and G are sequencing lanes generated by reverse transcription in the presence of ddATP, ddTTP, ddGTP, and ddCTP, respectively. Nucleotides are numbered according to their position in an RNA transcript. The triangles on the right side of the gels indicate increased base reactivity upon MtbCspB addition, whereas the inverted triangles indicate the opposite. Nucleotides are numbered according to the scheme of (**a**,**b**).

To localize the protein binding sites on the sRNAs, we used the chemical probing of MtbCspB/MTS0997 and MtbCspB/MTS1338 complexes (Figure 3). In case of MTS0997 sRNA, reactivity of nucleotides in the presence of MtbCspB increased, which means that the RNA structure changed and the bases became unpaired and solvent-exposed. Although some nucleotides are predicted to be unpaired, their reactivity may increase due to changes in the tertiary structure of RNA. Unlike MTS0997, MTS1338 nucleotides mainly accessible to chemical modification in a free state were less reactive to these probes in the presence of MtbCspB. Compared to the predicted secondary structure, most of the nucleotides available for chemical modification in the unbound state form base pairs, which is somewhat confusing. This may be due to the fact that such a large molecule as a 136-nucleotide RNA can exist in different states. We attempted to propose a secondary structure based on our chemical probing data (Figure 3c).

### 2.5. MtbCspB Can Bind to Short Variants of the RNAs

**Figure 4:**
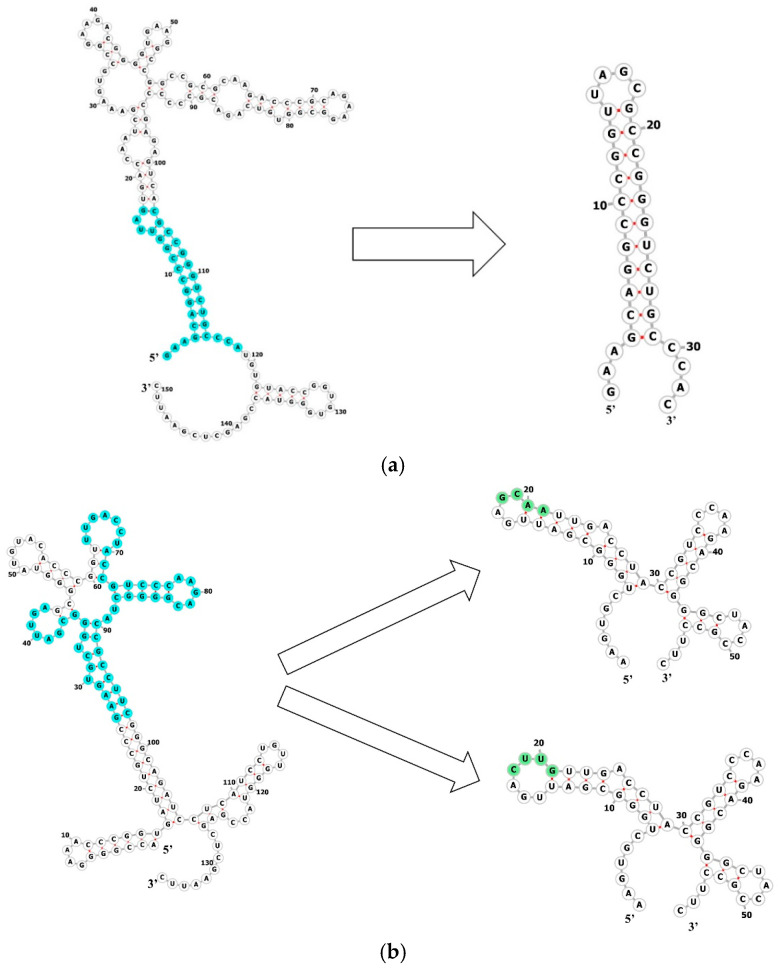
Secondary structure models of the RNA fragments: shortMTS0997 (**a**), shortMTS1338-GCAA and shortMTS1338-CUUG (**b**). Connecting sequences are shown in green. On the structure of the whole RNA, the RNA fragments are shown in cyan.

For further localization of an MtbCspB binding site on RNA, we obtained several RNA fragments. Based on the analysis of secondary structure models and the results of RNA chemical probing, we obtained one RNA fragment for MTS0997 (shortMTS0997) and two fragments for MTS1338 (shortMTS1338-GCAA and shortMTS1338-CUUG) (Figure 4).

SPR measurements (Table 2, Figure S3) showed that MtbCspB appeared to retain affinity for the RNA fragments, although the KD of MtbCspB complexes with MTS0997 and MTS1338 was two to three orders of magnitude lower (Table 1). Importantly, when using a buffer with a higher ionic strength (0.25 M NaCl instead of 0.15 M NaCl) MtbCspB retained the ability to bind to the RNA fragments but lost affinity to MTS0997 and MTS1338 sRNAs.

## 3. Discussion

### 3.1. Structure of the CspB from M. tuberculosis

In both Gram-negative and Gram-positive bacteria, stable sRNA:mRNA base pairing typically requires the assistance of RNA chaperones like Hfq or ProQ [10,24]. These proteins are also involved in many aspects of RNA life, such as refolding, annealing, protection, activation or inhibition of translation [10]. However, mycobacterium species have been reported to lack genes encoding Hfq and ProQ in their chromosome [12,25], which suggests the need to investigate how sRNAs in mycobacterial species acquire stability inside the cells [1]. Cold shock domain proteins (CSPs) are a potential candidate for this role. *M. tuberculosis* encodes two cold shock proteins, MtbCspA and MtbCspB. As shown previously, CspA from *M. tuberculosis* is a small protein with a single cold-shock domain [21]. In contrast, MtbCspB has an extension at the C-terminus, which is predicted to form two α-helices so that the N- and C-terminal domains are connected by a long flexible loop (Figure 2a). AlphFold3 predicted that the protein could form a dimer linked by α-helixes (Figure 2b). The crystal structure of MtbCspB confirmed the existence of a protein dimer connecting pairs of the C-terminal α-helixes, which form a stable four-helix bundle (Figure 2c,d). This bundle is a rather common ternary structure for α-helical proteins, for example, ROP/ROM proteins. A comparison of the MtbCspB model and the protein’s crystal structure approved the high mobility of the protein domains, as their positions in the model and the structure are differ. Furthermore, the connecting loop 71–84 lacked electron density in the crystal, further confirming high flexibility of the loop and independence of the MtbCspB domains’ positions in solution. It seems that this domain flexibility can also explain the broadening of the dimeric protein peak in gel filtration analysis performed under native conditions. Indeed, the difference in MtbCspB conformation would affect the radius of gyration of the protein molecule, which, in turn, would influence the time it takes for the protein to leave the column.

### 3.2. RNA-Binding Properties of the CspB from M. tuberculosis

In contrast with MtbCspA [21], this protein demonstrates remarkable affinity to RNAs. We dissected MtbCspB’s affinity for two sRNAs, which act as a key element for successful infection of its host—MTS0997 and MTS1338. Kinetic analysis has shown that these RNAs interact with MtbCspB dimers with a nanomolar dissociation constant (Table 2). The affinity of MtbCspB for the short RNA with six uridines at the 3′-end (oligoU RNA) is two orders of magnitude lower than for the studied sRNAs. The oligoU RNA is insufficient in length to bind to both parts of MtbCspB. Therefore, it can be hypothesized that the protein retains the ability to interact with oligoU RNA via the N-terminal cold shock domain, similar to its single-domain homologues. However, when MtbCspB bound to a large sRNA, the protein can interact with the protruding RNA via two N-terminal domains of the dimer, which significantly increases the affinity of MtbCspB for the RNA.

Chemical probing of the sRNAs in a complex with MtbCspB revealed that the reactivity of a number of nucleotides was changed (Figure 4). This suggests that MtbCspB might both stabilize single-stranded regions of RNAs and protect them from RNAse degradation. In the case of MTS1338 mRNA, MtbCspB protects nucleotides from modification, but in the case of MTS0997, nucleotide reactivity increases in the presence of MtbCspB. It is worth mentioning that the mature form of MTS1338 includes 109 nt [7], but in our experiments we used 117nt form of the sRNA. We found that most of the protected nucleotides are located at the 3′-end of MTS1338, so we could propose that MtbCspB may prevent sRNA maturation.

To determine the protein binding sites for the sRNAs, we designed three various RNA fragments that should retain affinity for MtbCspB based on the chemical probing data and prediction of the RNAs’ secondary structure (Figure 4). For MTS1338, the area of study was the stem at the 5′ end of the RNA, which was protected by MtbCspB. For MTS0997, the putative protein-binding site included nucleotides 29–48 and 65–100 from the original RNA sequence. GCAA or CUUG tetraloops were used to close the cutting edges of the RNA fragments. The affinity of MtbCspB for the RNA fragments was measured in buffer of different ionic strength, 150 mM and 250 mM NaCl. In a buffer with 150 mM NaCl, the affinity of MtbCspB for the RNA fragments was two orders of magnitude lower than for sRNA, as was the case for oligoU RNA. In a buffer containing 250 mM NaCl, the RNA fragments retain their ability to bind MtbCspB, whereas full-length sRNA does not bind to MtbCspB under these conditions. This unexpected result can be explained by the fact that high ionic strength causes dissociation of the MtbCspB dimers, resulting in loss of affinity to full-length RNAs, but the affinity of the protein monomer for short RNA remains unchanged. It can be assumed that MtbCspB in the dimeric form first recognizes the spatial structure of RNA and then binds to a specific nucleotide sequence. The results of the SPR showed that the affinity of the protein for RNA fragments is comparable to the affinity of the protein for oligoU RNA. It can be assumed that just the cold shock domain is involved in the interaction with RNA fragments. This may provide further evidence that the cold shock domain is sufficient for interaction with short RNAs, whereas dimer formation is required for interaction with long RNAs.

## 4. Materials and Methods

### 4.1. Gene Cloning, Expression and Purification of MtbCspB

The MtbCspB coding region was PCR-amplified from *M. tuberculosis* genomic DNA (strain H37Rv, kindly provided by Dr. T. L. Azhikina, Shemyakin and Ovchinnikov Institute of Bioorganic Chemistry RAS) and cloned into the expression vector pET28a (Invitrogen) using NcoI and HindIII sites. The pET28a vector carrying the MtbCspB gene was used for transformation in *E. coli* BL21(DE3)/pRARE to express a C-terminal His6-tag protein. Transformed cells were grown in LB medium containing 50 μg/mL kanamycin and 10 μg/mL chloramphenicol at 37 °C until the OD600 reached ~0.8. Protein expression was induced with 0.5 mM IPTG. After incubation for 3 h at 37 °C the cells were harvested by centrifugation at 8000 *g* for 20 min at 4 °C, resuspended in 40 mL lysis buffer (20 mM sodium phosphate buffer, pH 8.0, 0.5 M NaCl, 10 mM imidazole, 1 mM PMSF, 1 mM DTT, 0.1% Triton X-100) and disrupted by sonication (Fisher Scientific, Pittsburgh, PA, USA). Cell membranes and ribosomes were precipitated using stepwise centrifugation at 14,000 *g* for 20 min and at 330,000 *g* for 50 min, respectively. Cleared lysates were loaded on a Ni-NTA Agarose (Qiagen, Hilden, Germany) column equilibrated with 20 mM sodium phosphate buffer, pH 8.0, 0.2 M NaCl, 10 mM imidazole. The MtbCspB was eluted in a linear gradient of imidazole from 10 mM to 250 mM. Then protein-containing fractions were pooled and ammonium sulfate was added to a final concentration of 1.5 M. The solution was applied to Butyl-Sepharose (Cytiva, Marlborough, MA, USA) equilibrated with 50 mM Tris-HCl, pH 8.0, 1 M NaCl, 1.5 M ammonium sulfate. MtbCspB was eluted in a linear gradient of ammonium sulfate from 1.5 M to 0 M and NaCl from 1 M to 200 mM. The last step of purification was size-exclusion chromatography on HiLoad 16/60 Superdex 75 prep grade (GE Healthcare, Uppsala, Sweden), equilibrated with 50 mM Tris-HCl, pH 8.0, 200 mM NaCl. For crystallization experiments, the protein was lyophilized and then diluted with a size exclusion chromatography buffer to a final concentration of 18 mg/mL.

### 4.2. Analysis of Protein Particle Size by Gel Filtration

For this analysis, we used an Acta Basic system (Amersham Biosciences, Amersham, UK) with a Superdex 75 Increase column, 10/300 GL (Cytiva, Marlborough, MA, USA), preliminarily equilibrated with 200 mM NaCl, 50 mM Tris-HCl, pH 8.0. The protein sample (1 mg/mL, 100 μL) was injected into the column and eluted with velocity 0.5 mL/min. The calibration curve was modeled using the gamma subunit of archaeal initiation factor aIF2γ from *Sulfolobus solfataricus* (45.8 kDa), ribosomal protein L1 from *Thermus thermophilus* (24.8 kDa), isolated domain I of L1 from *T. thermophilus* (15.4 kDa) and CspB from *Bacillus subtilis* (7.3 kDa).

### 4.3. Protein Crystallization

Crystallization experiments were carried out at 25 °C using the vapor diffusion method with hanging drops on siliconized glass cover slides in Libro plates. MtbCspB formed crystals under condition #B5 of Nuc-Pro 3 (1.7 M lithium sulfate, 50 mM HEPES sodium salt, pH 7.0, 50 mM magnesium sulfate, Jena Bioscience, Jena, Germany) used as a reservoir solution. Drops were made by mixing the protein solution with the reservoir solution in 1:1 volume ratios. Crystals appeared after 3 weeks and grew within 1 week. Before freezing in liquid nitrogen, the crystals were transferred to the mother liquor containing 37.5% glucose.

### 4.4. X-Ray Diffraction Data Collection and Processing

Diffraction data were collected on a Rigaku XtaLAB Synergy-S single-crystal diffractometer of the Center for Collective Use “Structural and Functional Studies of Proteins and RNA”, Institute of Protein Research RAS (Pushchino, Russia), under the control of the CrysalisPro program (Rigaku, Akishima, Japan). The data were processed in space group P3121 using the CrysalisPro. The protein structure was determined by molecular replacement with PHASER (ver. 2.1.2) [26] using the AlphaFold model (AF-I6WZM9-F1) of MtbCspB (UNIPROT I6WZM9) as a starting point. The asymmetric unit of the crystal cells contained a protein monomer. The structures were refined using REFMAC (ver. 5.0.32) [27] from the CCP4 package (version 9) [28]. Manual editing and modification of the models were carried out using COOT (WinCoot 0.9.8.95) [29]. Statistics of data collection and crystallographic refinement are presented in Table 3.

### 4.5. Cloning and Purification of RNAs

The MTS0997 and MTS1338 coding regions were amplified by PCR from the *M. tuberculosis* (strain H37Rv) genomic DNA and cloned into the vector pUC18 (Invitrogen, Carlsbad, CA, USA) using HindIII and XmaI sites; the forward primers additionally contained sequence of the T7 promoter. DNA fragments corresponding to the sRNAs were obtained by PCR using pairs of overlapping primers. RNAs were obtained from linearized plasmid DNA by *in vitro* transcription with T7 RNA polymerase as described in [30]. RNA was purified on denaturing (8 M urea) 8% (*w*/*v*) acrylamide (19:1, acrylamide/bis-acrylamide) gels, using 40 mM Tris-acetate, pH 7.6, 1 mM EDTA, as running buffer. RNA was eluted by 50 mM Tris–HCl buffer, pH 7.5 (25 °C), 100 mM NaCl, purified by anion-exchange (DEAE-Sepharose) chromatography, precipitated by ethanol and dissolved in RNAse-free water.

### 4.6. RNA Chemical Probing

Structural analysis of sRNAs alone (MTS0977 and MTS1338) or in the presence of MtbCspB was performed using chemical probing of RNA as previously described [8]. For each RNA/protein combination chemical probing experiments were performed in triplicate.

#### 4.6.1. Chemical Modifications of RNA

For the chemical probing experiment, we used two types of samples: individual RNAs and RNAs mixed with MtbCspB. RNAs were heated to 80 °C for 10 min and cooled on ice before the addition of the protein. Then, RNAs (3 nM) were mixed with MtbCspB (6 nM) in a buffer containing 20 mM HEPES-NaOH, pH 8.0 and 150 mM NaCl. Samples were incubated at room temperature for 30 min. Control (free RNA) samples were incubated identically. Modification reactions were carried out in 50 μL of 20 mM HEPES-NaOH, pH 8.0 and 150 mM NaCl buffer containing RNA (750 pM) or RNA with MtbCspB. The modification was performed by addition of freshly prepared DMS (1 µL of a 1/5 dilution in ethanol), kethoxal (1 µL of a 1/5 dilution in ethanol), or CMCT (50 µL of 100 mg/mL in the reaction buffer) followed by incubation at 37 °C for 10 min. Reactions were stopped by adding of stop-buffers followed by ethanol precipitation. DMS stop-buffer contained 1 M Tris-HCl, pH 7.5, 1 M β-mercaptoethanol, and 100 mM sodium EDTA. CMCT stop-buffer contained 3 M sodium acetate. Kethoxal stop-buffer contained 150 mM sodium acetate and 250 mM H_3_BO_3_-KOH, pH 7.0. The RNAs were precipitated by ethanol and were washed with cold 70% ethanol. Pellets were dissolved in a buffer containing 25 mM H_3_BO_3_-KOH, pH 7.0, 300 mM sodium acetate, 0.5% SDS, and 5 mM sodium EDTA, followed by phenol–chloroform extraction and ethanol precipitation. RNA pellets were dissolved in 10 µL of 25 mM H_3_BO_3_-KOH, pH 7.0. Control (unmodified) samples were treated in the same way as modified samples, except that the modification step was skipped. Unmodified RNAs were used as templates for the reference sequencing reactions and to monitor artifact stops or pauses in reverse transcription.

#### 4.6.2. Fluorescent Primer Extension

RNA fragments used for chemical probing experiments contain identical 3′-ends, which correspond to the pUC18 sequence between restriction sites SmaI and EcoRI (5′-GGGUACCGAGCUCGAAUUC-3′). A Cy5 fluorescent reverse transcription primer complementary to the 3′-end of the studied RNA fragments was purchased from Evrogen (Moscow, Russia). For reverse transcription (RT), RNAs or modified RNAs (4 µL of a 1 µM solution) were added to the Cy5-primer (4 µL of a 2 µM solution) in an annealing buffer (250 mM Tris-HCl, pH 8.3, 200 mM KCl), heated to 90 °C for 3 min, and slowly cooled to 37 °C. RT reactions were performed in 50 mM Tris-HCl, pH 8.3, 40 mM KCl, 6 mM MgCl_2_, and 2 mM each dNTP. M-MuLV Reverse Transcriptase 5 units (SibEnzyme, Novosibirsk, Russia) were added and reactions were incubated at 37 °C for 60 min. For sequencing samples, 1 µM ddATP, ddCTP, ddGTP, or ddTTP was added to the RT reactions of each sample. Reactions were stopped by adding formamide with 10 mM EDTA, 0.3% bromophenol blue, and 0.3% xylenecyanol. Aliquots of 6–8 µL were analyzed in an 8% polyacrylamide gel in the presence of 8 M urea. Gels were visualized using the ChemiDoc MP Imaging System (Bio-Rad, Berkeley, CA, USA).

### 4.7. Analysis of MtbCspB-RNA Interaction

Kinetic analysis of protein interaction with specific RNA fragments was performed by surface plasmon resonance technique [31] using the imSPR-Pro system (iCLUEBIO, Seoul, Republic of Korea). MtbCspB was immobilized on the COOH chip (iCLUEBIO, Republic of Korea) and five different concentrations of the analyte samples (RNAs) were prepared by serial dilution in a solution containing 20 mM HEPES-NaOH, pH 8.0, 150 mM or 250 mM NaCl, 10 mM MgCl_2_ and 0.05% Tween-20 for each set of sensorgrams. The samples were injected at a flow rate of 30 µL/min. The injection step included a 300 s association phase followed by a 600 s dissociation phase in the buffer. Kinetic analysis was performed by globally fitting curves describing the simple 1:1 bimolecular model to a set of three to five sensorgrams using BIAEvaluation v. 4.1 software. As controls, biotinylated oligoU and oligoC were obtained as described [22]. Each immobilization strategy and kinetic analysis was repeated at least three times.

## 5. Conclusions

In most bacteria, so-called RNA chaperones—proteins that facilitate the interaction of sRNA with its target mRNA—play a key role in the functioning of small regulatory trans-coding sRNAs. The function of RNA chaperones in a number of Gram-negative and Gram-positive bacteria is actively studied, but their possible involvement in the regulation of gene expression in mycobacteria is virtually unknown. Orthologues of the most common RNA chaperones, Hfq and ProQ, have not been detected in *M. tuberculosis*; however, mycobacteria contain proteins with a cold shock domain, which are also involved in RNA remodeling in bacteria, and some of these possess RNA chaperone activity.

Our work is a step toward the discovery of an RNA chaperone in mycobacteria. We demonstrated for the first time that *M. tuberculosis* CspB forms a dimer due to its elongated C-terminal region, which is organized into a stable four-helix bundle. The protein exhibits high affinity for two different small RNAs from *M. tuberculosis* (KD is approximately 10^−10^ M), which is consistent with classical bacterial RNA chaperones. MtbCspB possesses a favorable combination of high affinity for RNA and an elongated structure with two RNA-binding monomers, making it an excellent candidate for an RNA chaperone in mycobacteria. *M. tuberculosis* CspB may be involved in the regulation of mycobacterial pathogenesis through interactions with small RNAs, but further research is needed to elucidate its role.

## Figures and Tables

**Table 1:** Kinetic constants and equilibrium dissociation constants (KD) of the MtbCspB-RNA complexes.

RNA	k_a_ (M^−1^ × s^−1^)	k_d_ (s^−1^)	K_D_ (nM)
MTS0997 sRNA	2.33 × 10^6^	2.62 × 10^−4^	0.112 ± 0.0056
MTS1338 sRNA	4.22 × 10^6^	1.25 × 10^−3^	0.296 ± 0.0148
oligoU RNA ^1^	1.18 × 10^4^	2.23 × 10^−4^	18.8 ± 0.94
oligoC RNA ^2^	not detected	not detected	not detected

^1^ 5′-GUGGUCAGUCGAGUGG-(U)6-3′. ^2^ 5′-GUGGUCAGUCGAGUGG-(C)6-3′.

**Table 2:** Kinetic constants and equilibrium dissociation constants (KD) of the MtbCspB-RNA fragment complexes in the buffer containing 150 mM or 250 mM NaCl (*).

RNA	k_a_ (M^−1^ × s^−1^)	k_d_ (s^−1^)	K_D_ (nM)
shortMTS0997	2.59 × 10^4^ 8.31 × 10^3^ *	1.59 × 10^−3^ 1.22 × 10^−3^ *	61.3 ± 3.06 147 ± 7.4 *
shortMTS1338-GCAA	2.19 × 10^4^ 8.72 × 10^3^ *	2.93 × 10^−4^ 5.11 × 10^−4^ *	13.3 ± 0.67 58.6 ± 2.93 *
shortMTS1338-CUUG	4.61 × 10^4^ 8.64 × 10^3^ *	1.82 × 10^−3^ 1.11 × 10^−3^ *	39.5 ± 1.98 128 ± 6.4 *

**Table 3:** Statistics of diffraction data processing and refinement of the MtbCspB structure.

PDB ID	9XW3
Wavelength, Å	1.5418
Resolution, Å	21.2–3.6 (3.7–3.6)
Space group	*P3*_1_21
Cell parameters, Å°	*a* = *b* = 91.57, *c* = 59.80; α = β = 90, γ = 120
Total number of reflections	26,010 (353)
Unique reflections	3524 (353)
Redundancy	7.4 (7.9)
Completeness, %	99.2 (100.0)
Mean *I*/σ(*I*)	9.95 (1.54)
*R*_merge(I)_	0.20 (0.73)
*R*_work_	0.22 (0.25)
*R*_free_	0.35 (0.30)
Number of protein atoms	950
Standard deviations	
bonds, Å	0.0057
bond angles, grad.	1.64
chiral volume	0.69
Ramachandran plot	
preferred, %	89.8
outliers, %	10.2
mean B-factor	100.2

Data in brackets corresponds to the highest resolution layer.

## Data Availability

The original contributions presented in this study are included in the article/Supplementary Material. Further inquiries can be directed to the corresponding author.

## Supplementary Materials

The supporting information can be downloaded at https://www.mdpi.com/article/10.3390/ijms27020663/s1.
