# Supplementary material for: The Cold Shock Protein CspB from Mycobacterium tuberculosis Binds to MTS0997 sRNA and MTS1338 sRNA as a Dimer
Source: Int J Mol Sci. 2026 Jan 9;27(2):663. doi: 10.3390/ijms27020663 (PMC12840758; doi:10.3390/ijms27020663)
Supplement: Supplementary file 1 [file ijms-27-00663-s001.zip › ijms-4061508-Supplementary Materials.pdf]

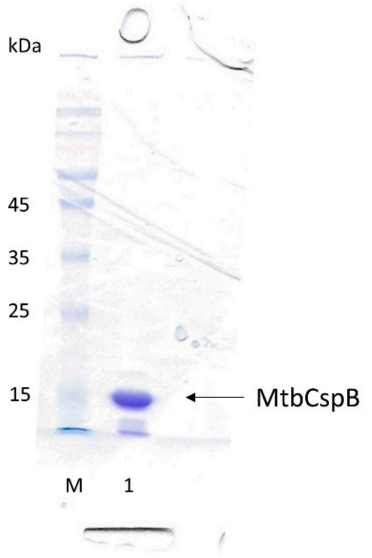

Figure S1. SDS-PAGE analysis of MtbCspB purity. Lane M – protein size marker, lane 1 – MtbCspB.

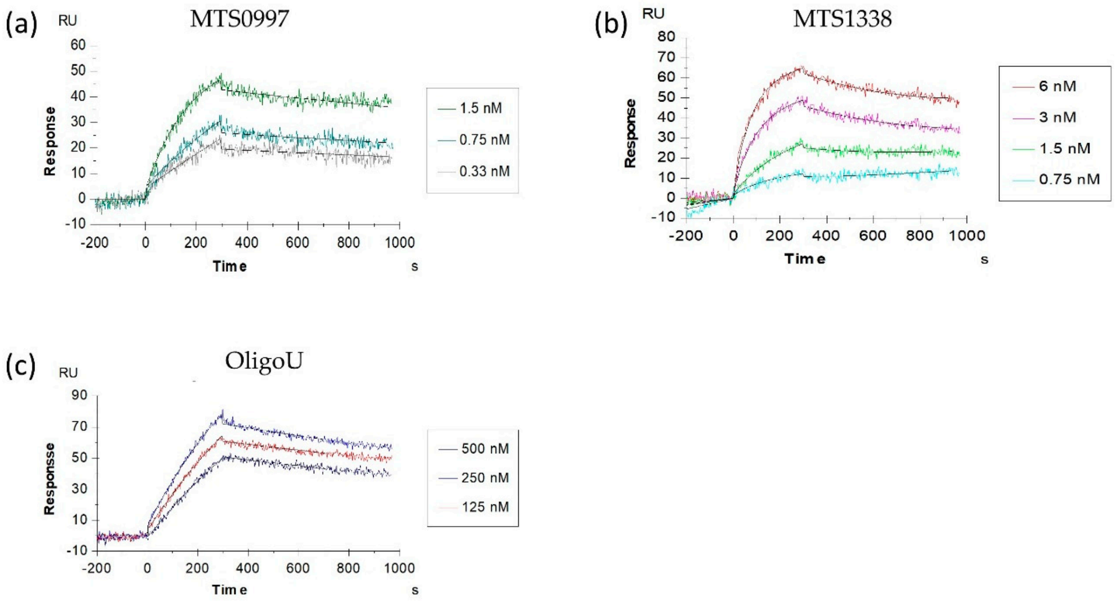

Figure S2. Sensograms showing binding kinetics for the CspB-RNA: (a) CspB with MTS0997 RNA (b) CspB with MTS1338 RNA; c) CspB with oligoU RNA. The analyte concentrations used for each data set are shown. Grey lines represent the global fit of the data sets using a 1:1 model.

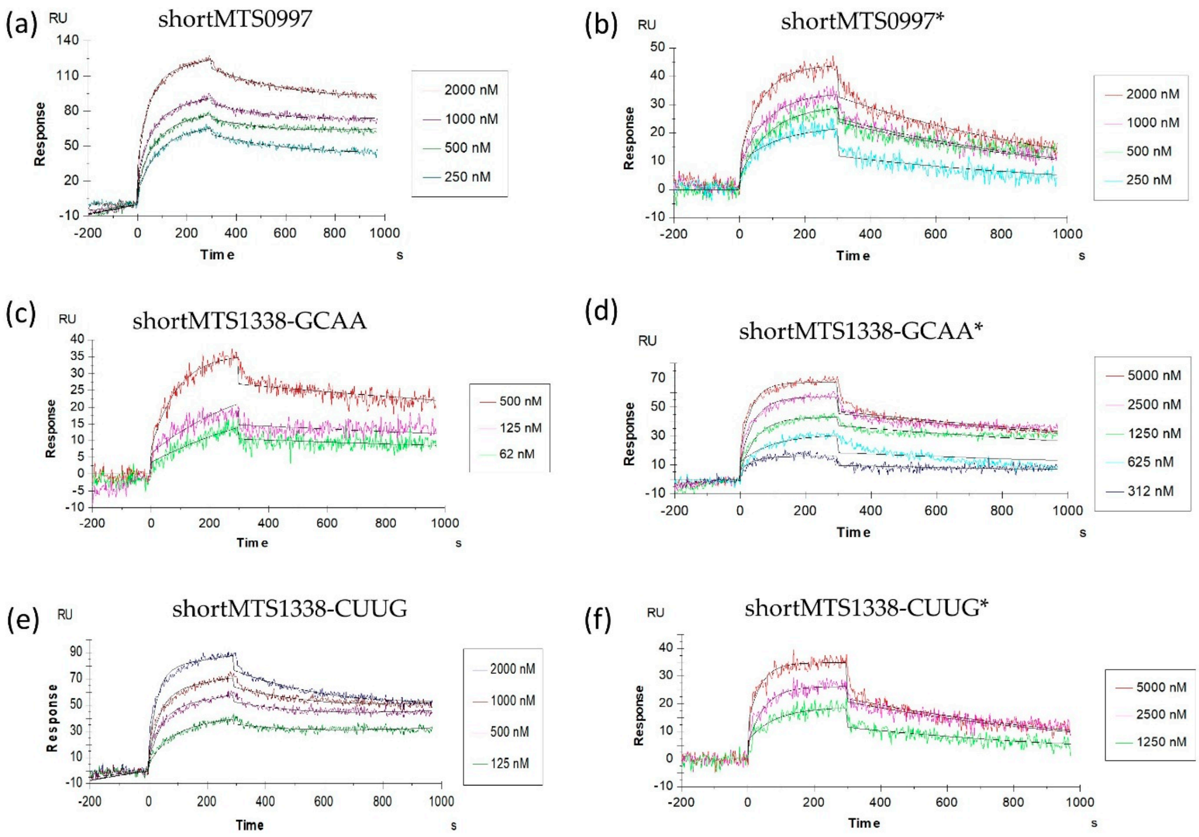

Figure S3. Sensograms showing binding kinetics for the CspB-RNA fragments: (a) CspB with shortMTS0997 RNA in the buffer containing 150 mM or (b) 250mM NaCl; c) CspB with shortMTS1338-GCAA RNA in the buffer containing 150 mM or (d) 250mM NaCl; (e) CspB with shortMTS1338-CUUG RNA in the buffer containing 150 mM or (f) 250mM NaCl. The analyte concentrations used for each data set are shown. Grey lines represent the global fit of the data sets using a 1:1 model.
